# Infection of Human Liver Myofibroblasts by Hepatitis C Virus: A Direct Mechanism of Liver Fibrosis in Hepatitis C

**DOI:** 10.1371/journal.pone.0134141

**Published:** 2015-07-27

**Authors:** Lynda Aoudjehane, Grégoire Bisch, Olivier Scatton, Christelle Granier, Jesintha Gaston, Chantal Housset, Philippe Roingeard, François-Loïc Cosset, Fabiano Perdigao, Pierre Balladur, Takaji Wakita, Yvon Calmus, Filomena Conti

**Affiliations:** 1 Sorbonne Universités, UPMC Univ Paris 06, UMR_S 938, CDR Saint-Antoine, F-75005, Paris, France; 2 INSERM, UMR_S 938, CDR Saint-Antoine, F-75012, Paris, France; 3 Human HepCell, Hôpital Saint-Antoine, F-75012 Paris, France; 4 AP-HP, Hôpital Pitié-Salpêtrière, Unité de Transplantation Hépatique, F-75013, Paris, France; 5 CIRI–International Center for Infectiology Research, Team EVIR, Université de Lyon, Lyon, France; 6 Inserm, U1111, Lyon, France; 7 Ecole Normale Supérieure de Lyon, Lyon, France; 8 Université Lyon 1, Centre International de Recherche en Infectiologie, Lyon, France; 9 CNRS, UMR5308, Lyon, France; 10 LabEx Ecofect, Université de Lyon, Lyon, France; 11 Institut Cochin, Université Paris Descartes, CNRS (UMR 8104), INSERM U1016, Paris, France; 12 INSERM U966, Université François Rabelais and CHRU de Tours, France; 13 AP-HP, Hôpital Saint Antoine, Département de la chirurgie digestive, F-75012, Paris, France; 14 National Institute of Infectious Diseases, Department of Virology II, Tokyo, Japan; SAINT LOUIS UNIVERSITY, UNITED STATES

## Abstract

**Background:**

Chronic hepatitis C is a major cause of liver fibrosis and cirrhosis. It is generally accepted that inflammation that occurs in response to hepatocyte infection by the hepatitis C virus (HCV) is the main mechanism that triggers myofibroblast differentiation and stimulation in chronic hepatitis C. The aim of this study was to determine if HCV might infect human liver myofibroblasts (HLMF) and directly stimulate their fibrogenic activities.

**Methods:**

We evaluated the expression of the viral entry receptors, levels of HCV-RNA and HCV-protein and the expression of fibrosis markers in HLMF by using quantitative PCR, western blot and immunofluorescence analyses. Pseudoparticles (HCVpp) and cell culture–derived HCV (HCVcc) were used to study the ability of HLMF to support viral entry, replication and fibrosis induction.

**Results:**

We showed that HLMF expressed all known molecules of the HCV receptor complex, *i*.*e*. CD81, LDL-R, scavenger receptor-BI, claudin-1 and occludin. These cells were also permissive to HCVpp entry. Inoculation with HCVcc caused short-term infection of these cells, as shown by their content in positive- and negative-strand HCV RNA, in core and NS3 viral proteins, and by their release of core protein levels in the culture supernatants. HCV infection stimulated myofibroblastic differentiation, proliferation and collagen production in these cells. In addition, evidence of *in vivo* infection was provided by the detection of positive- and negative-strand HCV RNA in preparations of HLMF obtained from HCV-infected patients.

**Conclusion:**

These findings indicate that HCV infection of HLMF can occur and trigger extracellular matrix overproduction, thereby contributing to the development of HCV-related liver fibrosis.

## Introduction

Hepatitis C virus (HCV) infection is the main cause of chronic liver disease, leading to progressive hepatic fibrosis and ultimately cirrhosis. Liver fibrosis is characterized by an accumulation of extracellular matrix (ECM) that leads to a distorted architecture and functional impairment of liver tissue [1]. The source of ECM production, including collagens, in the injured liver are myofibroblasts, the origins of which are diverse and mainly represented by hepatic stellate cells and portal mesenchymal cells [2]. In a context of chronic liver injury, these different cell types acquire myofibroblastic features such as alpha-smooth muscle actin (a-SMA) expression, become proliferative and overproduce constituents of the ECM. It is currently assumed that the persistent damage of hepatocytes caused by HCV infection triggers myofibroblast differentiation and stimulation *via* the recruitment and activation of inflammatory cells in the liver [3]. Injured hepatocytes and their neighboring sinusoidal cells (*e*.*g*. Endothelial cells and Kupffer cells) can all release pro-inflammatory cytokines [4,5].

However, HCV infection does not consistently induce marked inflammation [6], particularly in fibrosing cholestatic hepatitis C, a highly aggressive course that can occur after liver transplantation, when mononuclear inflammation is moderate [7]. Studies of cultured cells and transgenic mice have indicated that HCV is not directly cytopathic, and that viral replication can occur in the absence of an overt inflammatory reaction [8], suggesting that HCV may interact directly with fibrocompetent cells to induce liver fibrosis. This is of mandatory importance, since fibrocompetent cells could be the target of antifibrotic drugs, that could benefit patients with a rapidly developing fibrotic process. Better understanding of the HCV replicative cycle, and of the fibrotic process induced by HCV, is thus needed in order to propose such new therapeutic strategies.

HCV has been previously shown to infect monocytes, lymphocytes and other cell types [9–11], in addition to hepatocytes [12]. HCV receptors include CD81 tetraspanin [13], low-density lipoprotein receptor (LDLR) [14], scavenger receptor class B type I (SR-BI), claudine-1 (CLDN-1) and occludin (OCLN), which are required for the entry of HCV into target cells [15,16]. The expression of CD81 and LDLR in human liver myofibroblasts (HLMF) has previously been reported [17], but the expression status of other receptors in these cells is unknown.

During this study, we examined the expression of several HCV candidate receptors in HLMF. We also showed that HLMF were permissive to pseudoparticles (HCVpp) entry. To test the ability of HCV to infect and stimulate these cells, we exposed them to cell-culture-derived HCV (HCVcc) obtained from the Huh7.5 cell line infected with the full-length genome of JFH-1, which is a genotype 2a HCV isolate with highly efficient genome replication [18]. We finally analyzed HLMF obtained from HCV-infected subjects.

## Materials and Methods

### Ethics statement

Liver tissue samples were taken in course of routine work and were obtained from surgical procedures carried out at the APHP Hospital, Paris, France. The consent for all samples was obtained. All samples were anonymized and no-opposition statement was given to the patients and signed by surgeon. This procedure was approved and granted by the Persons Protection Comittee (CPP Ile de France III) and by the French Ministry of Health (Ref COL 2929 and COL 2930)

### Cell culture models

Liver tissue samples were obtained from HCV-infected and uninfected subjects undergoing partial hepatectomy. Normal non-infected liver tissue was obtained from 15 adult patients undergoing partial hepatectomy for the treatment of colorectal cancer metastases and all were seronegative for HCV, HBV and HIV. The patients were aged 55±6 years (mean±DS), and included 10 males and five females whose histological evaluation was considered as normal. Two HCV-positive male patients were also evaluated; they were undergoing partial hepatectomy for hepatocellular carcinoma, were aged 58±4 years, and their serological findings were negative for HBV and HIV. These two patients were HCV genotype 1 and their viral loads were 6.5 and 5.5 log, respectively.

The procedures for cell isolation were carried out in compliance with French ethical guidelines, using established methods [19]. The liver tissue fragments were perfused, first with Liver Perfusion Medium (Invitrogen, Cergy Pontoise, France) at 37°C for 15 min, and then with collagenase and dispase-containing Liver Digest Medium (Invitrogen) at 37°C for approximately 15 min, until the tissue was completely digested. Cells were dispersed by gentle shaking, filtered and centrifuged at a low speed. Primary human hepatocytes (PHH) were collected from the pellet and used in primary culture, as previously described [12]. The non-parenchymal cell fraction was collected from the supernatant which was centrifuged to obtain hepatic liver myofibroblasts (HLMF), as described elsewhere [19]. After 1 week of primary culture and two passages, all the cells had a myofibroblast-like appearance and stained positive for vimentin and alpha-smooth muscle actin. In most experiments, cell preparations from at least seven different individuals were used after 2 passages. In some experiments, cells obtained at passage 3 to 6 were used. The 3T3 mouse fibroblast, the 293T human embryonic kidney and the HepG2 human hepatocellular carcinoma cell lines were obtained from ATCC (Molsheim, France). The T98G human glioblastoma multiforma tumor was kindly supplied by Christine Granotier (Laboratoire de Radiopathologie, UMR967, Fontenay-aux-Roses).

### Characterization of HLMFs

The expressions of CD90 (fibroblasts), CD31 (endothelial cells), CD14 (macrophages), a-SMA (myofibroblasts), vimentin (mesenchymal cells) and the hepatocyte marker (human-specific hepatocyte marker, code M7158; Dako) were measured by flow cytometry analysis. HLMFs were released from culture plates by trypsination, centrifuged, re-suspended in PBS (3**x**10^5^ cells per ml) and then washed. The cells were then stained with blue LIVE/DEAD cell viability dye for 30 minutes (Invitrogen), according to the manufacturer's instructions. After washing with PBS, the HLMFs were fixed with 4% paraformaldehyde (PFA). A multicolor flow cytometry using antibodies with directly-conjugated fluorochromes was used, and the cells were stained with a cocktail of conjugated anti-membrane antibodies: phycoerythrin (PE)-conjugated anti-human CD31 (dilution 1/20; Beckman Coulter, France), Allophycocyanin-Alexa-fluor 750 (APC alexa-fluor-750)-conjugated-anti-human CD14- (dilution 1/20; Beckman Coulter), phycoerythrin-Cy5 (PC5)-conjugated anti-human CD90 (dilution 1/20; Beckman Coulter), or with a cocktail of isotypic antibodies specific to each antibody used (dilution 1/20; Beckman Coulter) for 45 min at 4°C. The cells were then permeabilized with BD Cytofix/Cytoperm solution (BD Biosciences, Le Pont De Claix, France) for 20 min and stained with a non-conjugated anti-humana-SMA (dilution 1/50; Dako), for 45 min at 4°C. The cells were then incubated with fluorescein isothiocyanate (FITC)-labeled goat anti-mouse IgG (dilution 1/100; Caltag, Burlingame, CA, USA) or a FITC-conjugated isotype (mouse IgG) as a negative control for 30 min at 4°C.

In parallel, for single-intracellular staining of vimentin (Dako), a-SMA or the specific hepatocyte marker (M7158, Dako), the cells were fixed and permeabilized with BD Cytofix/Cytoperm solution (BD Biosciences, Le Pont De Claix, France) for 20 min and then stained with non-conjugated anti-humana-SMA or vimentin or the hepatocyte marker (dilution 1/50; Dako), for 45 min. The cells were then incubated with PE-labeled goat anti-mouse IgG (dilution 1/100; Caltag, Burlingame, CA, USA) or a PE-conjugated isotype (mouse IgG) as a negative control for 30 min. Cell staining was then determined using a BD LSR II flow cytometer and analyzed using FACSDiva software (Version 6.1.1, BD Biosciences).

### Production of HCVpp and Infection Assays

The expression vector for the E1E2 glycoproteins of HCV strain H77 (1a; phCMV-deltE1E2 (H77) and HCV strain JFH-1 (2a; phCMV-delta-JFH1) were described previously [20]. 293T cells were transfected with a HIV-1 Gag-Pol packaging construct, an PWPTSnls-LacZ-based transfer vector encoding a nuclear-target ß-galactosidase, and the E1E2 expression constructs, VSV envelope glycoprotein G (phCMV-VSV-G) or empty vector (pUC19 no envelops). The medium was changed 16h after transfection. Supernatants containing the pseudo-particles were harvested 24 h later, filtered and used in infection assays.

For infection assays, Target Huh7.5, PHH and HLMF cells were seeded in 12-well plates and incubated overnight at 37°C for 24h. Viral supernatants containing the pseudo-particles were added to the cells and the plates were incubated overnight. The supernatants were removed and the cells were incubated in new medium for 72 h at 37°C.

### Detection of pseudo-particles

The expression of LacZ in cultured cells was evaluated with X-Gal Staining as described previously [20]. First, the cells were washed with cold PBS and fixed with glutaraldehyde for 10 min at room temperature. Then, the cells were incubated with X-Gal solution at 37°C for 1–16 hours, depending on staining intensity checked every hour under a light microscope. The infectious titers were expressed as transducing units (TU) per milliliter.

### Inhibition of HCVpp entry

Huh7.5, PHH and HLMF cells were pre-incubated with 10 μg/ml anti-CD81 or irrelevant IgG control mAb. After 1 hour, HCV-H77pp, HCVpp-JFH1, VSV-Gpp, or No-envpp was added and the cells incubated overnight. The supernatants were removed and the cells were incubated in new medium for 72 hours at 37°C. The expression of LacZ in cultured cells was assessed using X-Gal staining and the percentage of positive cells measured.

### HCVcc inoculation and inhibition studies

Highly permissive subclones of the human hepatocellular carcinoma-derived Huh-7 have been shown to produce workable titers of cell-culture-derived virus (HCVcc) when replicating the full-length genome of JFH1 [21]. High-titer stocks of JFH1-HCVcc were prepared from Huh7.5 cells (a kind gift from Wakita Takaji, Tokyo, Japan), as previously described [12]. The infectivity titer was expressed in focus-forming units (ffu) per ml of supernatant. JFH1-HCVcc stocks or UV-inactivated HCVcc, as previously described [22,23] were used to inoculate HLMF, 24 hours after plating, at a multiplicity of infection (MOI) of 0.1 ffu per cell. After 12-hour incubation, the inoculum was removed and the cells were washed. The cultures were then continued in complete medium for a maximum of 8 days, after which HLMF became confluent and started to undergo cell death (S2 Fig). The 3T3 and 293T cell lines were used as negative controls of cell infection, and Huh7.5 and PHH were used as positive controls (S3 Fig).

For inhibition studies, HLMF were pre-incubated with increasing concentrations of an anti-CD81 neutralizing monoclonal antibody or an isotype-matched control antibody (Isotype mouse IgG1, k) (Both from BD Farmagen, Le Pont De Claix, France) for 1 h before HCVcc inoculation, or interferon-alpha (IFN-a after HCVcc inoculation. HCV infection was evaluated three or eight days after the challenge. For inhibition studies, Huh7.5 and PHH were used as controls (S4 Fig).

### Real-Time Quantitative PCR for the detection of HCV, HCV receptors, and fibrosis markers

Total RNA was extracted from cultured cells or from filtered culture supernatants, using the RNeasy and Qiamp viral RNA mini kits (Qiagen SA, Courtaboeuf, France). RNA for human gene expression was subjected to reverse transcription using random hexamer primers (Invitrogen) and avian myeloblastosis virus reverse transcriptase (Promega, Charbonnières, France). HCV RNA was quantified in cells and culture supernatants using a strand-specific reverse real-time PCR technique previously described [24]. The primer sequences used for HCV and human genes are shown in Table 1. Quantitative real-time PCR was performed using the DNA Fast Start Sybr Green kit, on a LightCycler device (Roche Diagnostics, Meylan, France).

**Table 1:** Primers used for quantitative real-time RT-PCR.

Gene	Accession number	Sequence of forward (F) primers	Sequence of reverse (R) primers	Size (bp)
Albumin (ALB)	NM_000477.5	5'-AGGGAGGTCTGGGCTATCATC-3’	5’-TTCGTGAAACCTATGGTGACATG-3’	101
CD81	NM_004356.3	5’- CCCCAAGGATGTGAAGCAGTTC-3'	5’-TCCCGGAGAAGAGGTCATCGAT-3'	245
CLDN1	NM_021101.4	5’-TTCTCGCCTTCCTGGGATG-3'	5’-CTTGAACGATTCTATTGCCATACC-3'	405
Collagen I (COL1A1)	NM_000088.3	5’-CCTCAAGGGCTCCAACGAG-3'	5’-TCAATCACTGTCTTGCCCCA-3’	116
Collagen IV (COL4A1)	NM_001845.4	5’-ATGTCAATGGCACCCATCAC-3’	5’-CTTCAAGGTGGACGGCGTAG-3’	363
CYP2E1	NM_000773.3	5’-AGCACAACTCTGAGATATGG-3’	5’-ATAGTCACTGTACTTGAACT-3’	366
GAPDH	NM_002046.3	5’-ACAGTCCATGCCATCACTGCC-3’	5’-GCCTGCTTCACCACCTTCTTG-3’	266
HNF-1b	NM_001165923.1	5’-GAAACAATGAGATCACTTCCTC-3’	5’CTTTGTGCAATTGCCATGACTC-3’	274
HCV		104S: 5’-AGAGCCATAGTGGTCTGCGG-3’	197R: 5’-CTTTCGCGACCCAACACTAC-3’	150
LDLR	NM_001195803.1	5’- CAATGTCTCACCAAGCTCTG-3'	5’-TCTGTCTCGAGGGGTACCTG-3'	258
OCLN	NM_001205254.1	5’-TGCATGTTCGACCAATGC-3’	5’-AAGCCACTTCCTCCATAAGG-3’	235
a-SMA	NM_001613.2	5’-TGAAGAGCATCCCACCCT-3’	5’-ACGAAGGAATAGCCACGC-3’	309
SR-B1 (SCARB1)	NM_005505.4	5’-CGGAATTCAGGGGTGTTTGAAGGC-3'	5’-CGGGATCCTGAATGGCCTCCTTATCC-3'	610
28S (RN28S1)	NR_003287.2	5’-TTGAAAATCCGGGGGAGAG-3’	5’- ACATTGTTCCAACATGCCAG-3’	100

### Immunodetection of HCV receptors and proteins

#### Flow cytometry

CD81 and LDL-R expression was assessed using flow cytometry with a BD LSR II flow cytometer and then analyzed using Diva (Version 6.1.1, BD Pharmagen). The cells were first fixed with 4% paraformaldehyde, then stained with non-conjugated antibodies: mouse anti-human CD81 (BD Pharmagen; 1/50) or rabbit anti-LDLR polyclonal antibody (Abcam, Cambridge, UK; 1/10), for 30 min. This was followed by incubation with PE-labeled goat anti-mouse IgG (dilution 1/100, BD Sciences) or FITC-labeled goat anti-rabbit IgG (dilution 1/100; abcam) for 30 min. During the assay, appropriate controls (PE-isotype IgG and FITC-isotype IgG) were always used to quantify background fluorescence. Huh7 and PHH were used as positive controls.

#### Indirect Immunofluorescence

Expression of all study receptors (CLDN1, OCLN, SR-BI, LDLR, CD81), a fibroblast-specific membrane marker (CD90) and HCV core protein expression were assessed using indirect immunofluorescence. Cells were seeded onto glass coverslips in 12-well plates at a density of 5x10^4^ cells per well. The cells were then fixed for 72h post-infection or post-plating (controls) in 4% paraformaldehyde, and then permeabilized (for intracellular staining) or not (extracellular staining) with 0.3% Triton X-100 in PBS, and incubated in 1% BSA/10% goat serum. The cells were then incubated overnight with the primary antibody (or an isotype-matched control antibody) at 4°C: mouse anti-human CD81 (clone JS-81, BD Pharmingen; 1/50), rabbit anti-SR-BI (Abcam; 1:50), rabbit anti-CLDN1 (Invitrogen; 1/50), mouse anti-OCLN (Invitrogen; 1/50) and rabbit anti-LDLR (Progen Biotechnik; Germany;1/50). PHH and Huh7.5 cells were used as positive controls.

To detect core protein, co-staining was performed on HLMFs, infected or not, with a rabbit anti-CD90 (Novus Biologicals, Cambridge, UK; 1/50) and mouse anti-Core (C7-50; Alexis Biochemicals, CA, USA; 1/50) or on Huh7.5, infected or not, with a rabbit anti-CLDN1 (1/50) and the same mouse anti-Core (1/50). The secondary antibodies were goat-anti-mouse or goat-anti-rabbit AlexaFluor488- or AlexaFluor594-conjugates (Invitrogen;1:200). Nuclei were detected using Dapi (1/1000 in PBS, Invitrogen). Images were captured with a Leica DMR inverted microscope using LAS image analysis.

#### Western blot analysis

Western blotting for SR-BI, CLDN1, OCLN, HCV nonstructural protein 3 (NS3) and HCV core protein was performed as previously described [12,19]. Anti-SR-BI polyclonal antibody (Abcam) and monoclonal antibodies against CLDN-1 (Zymed, San Francisco, CA, USA), OCLN (ABCYS, Paris, France), HCV core protein (C7-50; Alexis Biochemicals, San Diego, CA, USA), NS3 (1847; Virostat, Portland, MA), or alpha-actin (Cedarlane Laboratories Ltd, Ontario, Canada) were used as primary antibodies.

### HCV core Ag quantification

Secreted HCV core protein was quantified in the filtered supernatant using an automated chemiluminescent microparticles immunoassay (Architect HCV Ag Test, Abbott, France) [25].

### Cell proliferation assay

HLMF were plated in 96-well plastic dishes (10^4^ cells/well) and inoculated with HCVcc, 24 hours after plating, as described above. After different times in culture, the cells were pulsed with [^3^H]-thymidine (1 μCi/well) for 16 hours. The cells were then harvested and radioactivity was counted. Cell death and viability after infection are presented in S2 Fig.

### Collagen immunoassay

The C-terminal propeptide of type I collagen was measured in the supernatant of HLMF using an enzyme immunoassay as previously described [26] (MicroVue CICP EIA kit, QUIDEL, San Diego, CA, USA) according to the manufacturer’s protocol. The dye concentration was estimated by spectrophotometry at 405 nm.

### Statistical analysis

Results are expressed as means ± SD, and differences between groups were tested by one-way ANOVA and the Mann-Whitney test implemented with Stat View IV software (Abacus Concepts, Berkeley, CA, USA). Differences with p values <0.05 were considered to be statistically significant.

## Results

### Characterization of HLMFs

**Fig 1:**
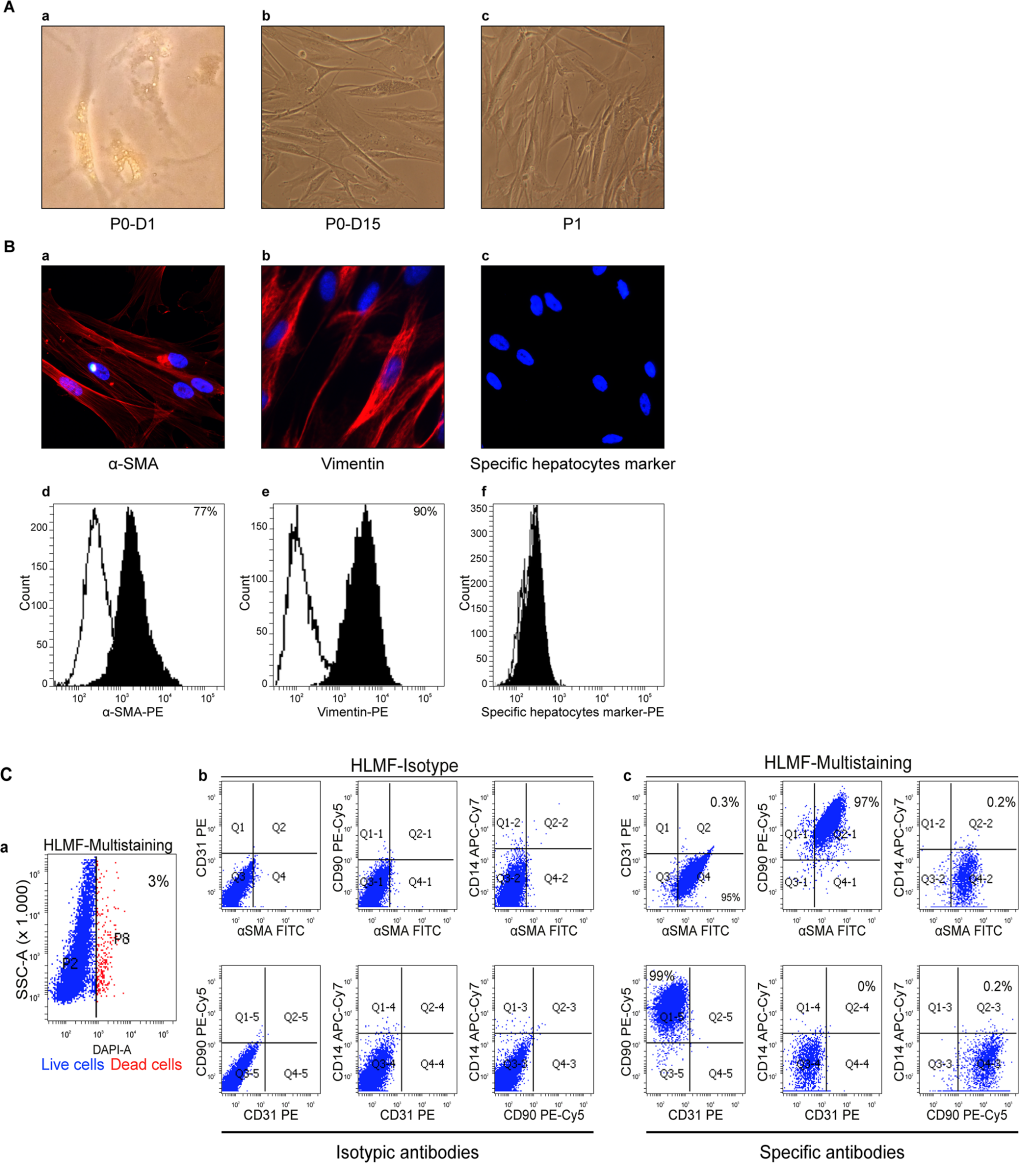
Characterization of HLMFs. **(A)** Morphological characteristics of HLMFs in culture at passage 0-Day 1 (magnification x40), passage 0 (A-a), Day 15 (A-b) and passage 1 (A-c) after isolation (magnification x20), shown by optical microscopy. **(B)** Single immunostaining by immunofluorescence and flow cytometry. HLMFs were stained with anti-a-SMA (B-a and-d), anti-vimentin (B-b and-e) and anti-specific hepatocyte marker (B-c and-f) antibodies. **(C)** Detection of CD90, CD14, CD31, a SMA, on HLMFs using multi-labeling by flow cytometry. HLMFs were stained with a cocktail of mouse monoclonal antibodies or negative controls (cells stained with the isotype control antibody) and then analyzed by flow cytometry. Histograms show the fluorescence intensity of the population after the exclusion of dead cells (C-a) and isotype control antibody (C-b) compared with the indicated marker (C-c) on HLMFs. The data from a single experiment are presented and are representative of all experiments used in this study

At isolation, up to 30% of the cells displayed autofluorescent retinoid droplets that were characteristic of hepatic stellate cells (Fig 1A-a), and after 1 week of primary culture through several passages (Fig 1A-b and-c), all the cells had a myofibroblast-like appearance and stained positive for vimentin and-smooth muscle actin and negative for the specific hepatocyte marker (Fig 1B).

To characterize and evaluate HLMFs, we performed a 4-color flow cytometric analysis. The gating used to separate the various cellular subsets is shown for representative populations circled in blue. The cell population after the exclusion of dead cells (Fig 1C-a) was gated according to its isotype control (Fig 1C-b). In these analyses, the majority of cells were CD90^+^ CD31^-^ CD14^-^ and a SMA^+^ (99%, 0%, 0% and 95%, respectively) (Fig 1C-c), indicating that HLMFs isolated using the method described had not been substantially contaminated by other cell types.

Hepatocyte contamination was always excluded using RT-PCR for the detection of albumin, CY2E1 and HNF-1 mRNAs, which are critical for hepatocyte markers. mRNA was always negative in our HLMFs (data not shown). During the cell characterization by flow cytometry, we always performed staining with an anti-hepatocyte-specific marker (M7158, Dako), and the results were consistently negative (data not shown).

### Expression of HCV entry factors in HLMF

**Fig 2:**
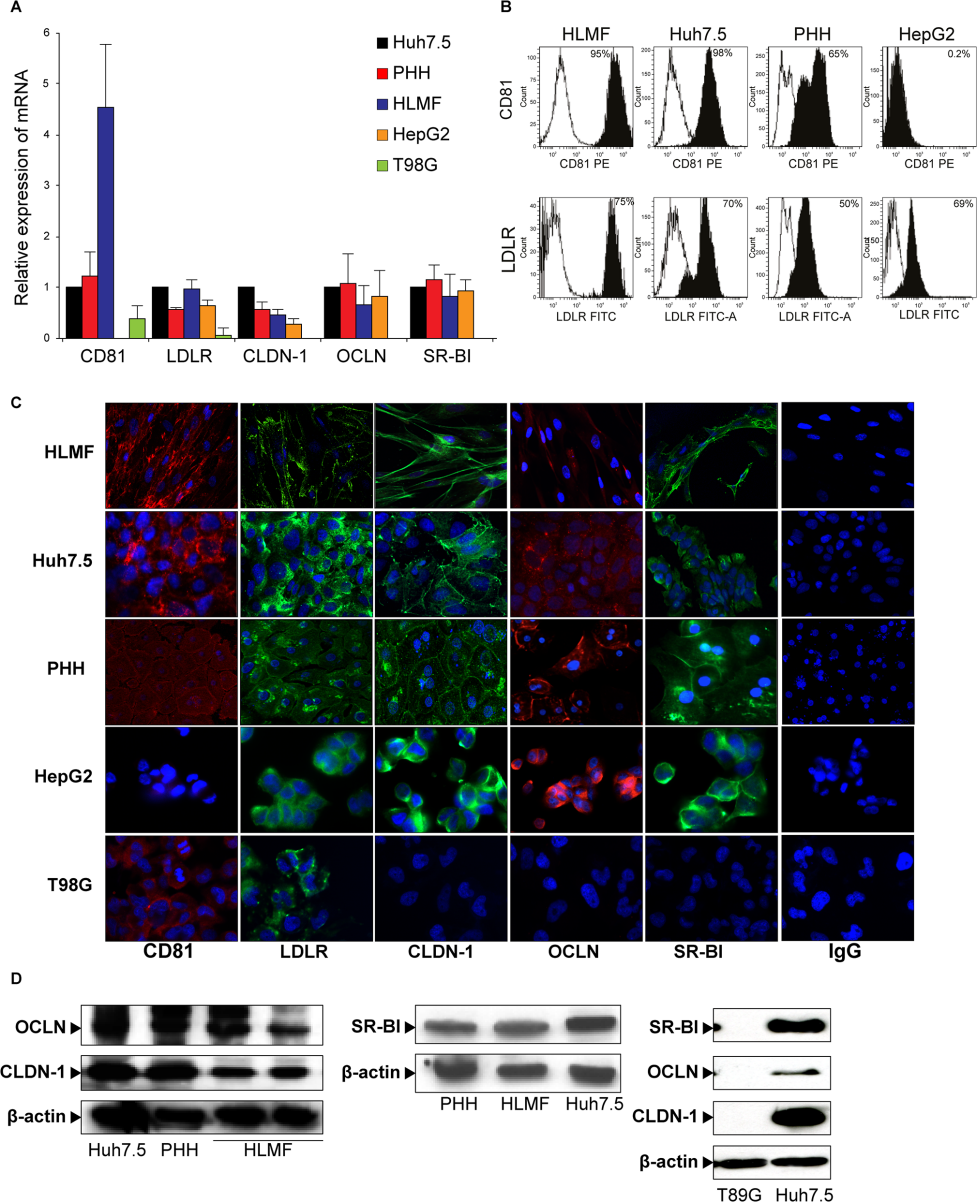
Expression of HCV entry receptors in human liver myofibroblasts (HLMF). HLMF were subjected to the analysis of HCV entry receptors (CD81, LDLR, CLDN-1, OCLN and SR-BI) using four techniques. **(A)** Quantitative RT–PCR. Histograms represent mRNA levels relative to Huh7.5 in PHH, HLMF, HepG2 and the T98G cell line (means ± SD from 7 cell preparations). **(B)** Flow cytometry (CD81 and LDLR). The histograms of mean fluorescence showed that Huh7.5, PHH and HLMF expressed CD81 and LDLR, whereas HepG2 expressed LDLR but not CD81. Appropriate IgG controls were used as negative controls. **(C)** Immunofluorescence. HLMF, Huh7.5, PHH, HepG2 and the T98G line were stained for HCV receptors and imaged with a Leica DMR inverted microscope using LAS image analysis (Original magnification X40). The Flow cytometry and IF results are representative of seven HLMF preparations. **(D)** CLDN-1, OCLN and SR-BI detection using Western blot analysis. Huh7.5 and PHH were used as positive controls and T98G as a negative control for all receptors. The blots are representative of three HLMF preparations.

CD81 and LDLR expression was evaluated by RT-PCR, flow cytometry and immunofluorescence, and expression of the newly identified HCV co-receptors CLDN-1, OCLN and SR-BI using RT-PCR, immunofluorescence and Western blot. The mRNAs of CD81, LDLR, CLDN-1, OCLN and SR-BI, evaluated by RT-PCR, were expressed in HLMF. The results obtained in cells at passage 2 are shown in comparison with Huh7.5 cells and PHH, used as positive controls and HepG2 and T98G cells, as negative control (Fig 2A). Flow cytometry was also used at passage 2 and 3 to detect CD81 and LDLR. We observed that 95% of HLMF were CD81-positive compared with 98% of Huh7.5, 65% of PHH and 0.2% of HepG2, and that 75% of HLMF were LDLR-positive compared with 70%, 50% and 69% of Huh7.5, PHH and HepG2, respectively. No staining was detected with the isotype-matched control antibody (Fig 2B).

Using immunofluorescence, we observed that the expression of HCV entry factors (CD81, LDLR, CLDN-1, OCLN and SR-BI) was detected in 40 to 90% of HLMF, comparable to what was observed in PHH and Huh7.5 cells (Fig 2C). T98G displayed no expression of CLDN-1, OCLN or SR-BI and only weakly expressed CD81 and LDLR. CD81 was not detected in HepG2 cells. No staining was detected with the isotype-matched control antibody (Fig 2C).

CLDN-1, OCLN and SR-BI proteins were also detected by western blotting in HLMF as in Huh7.5 and PHH (positive controls) (Fig 2D). As anticipated from previous work [27], the T98G cell line did not express the receptors.

Thus, most receptors known to bind HCV at the cell surface before entry were expressed in HLMF. Importantly, the expression of HCV entry factors underwent noticeable changes in HLMF along successive passages: OCLN and LDLR expressions, evaluated by RT-PCR, strongly decreased from passage 4 to passage 6, while that the expression of CD81 increased form passage 2 and then remained stable between other passages studied (S1 Fig).

### Infection of HLMF by HCVpp

**Fig 3:**
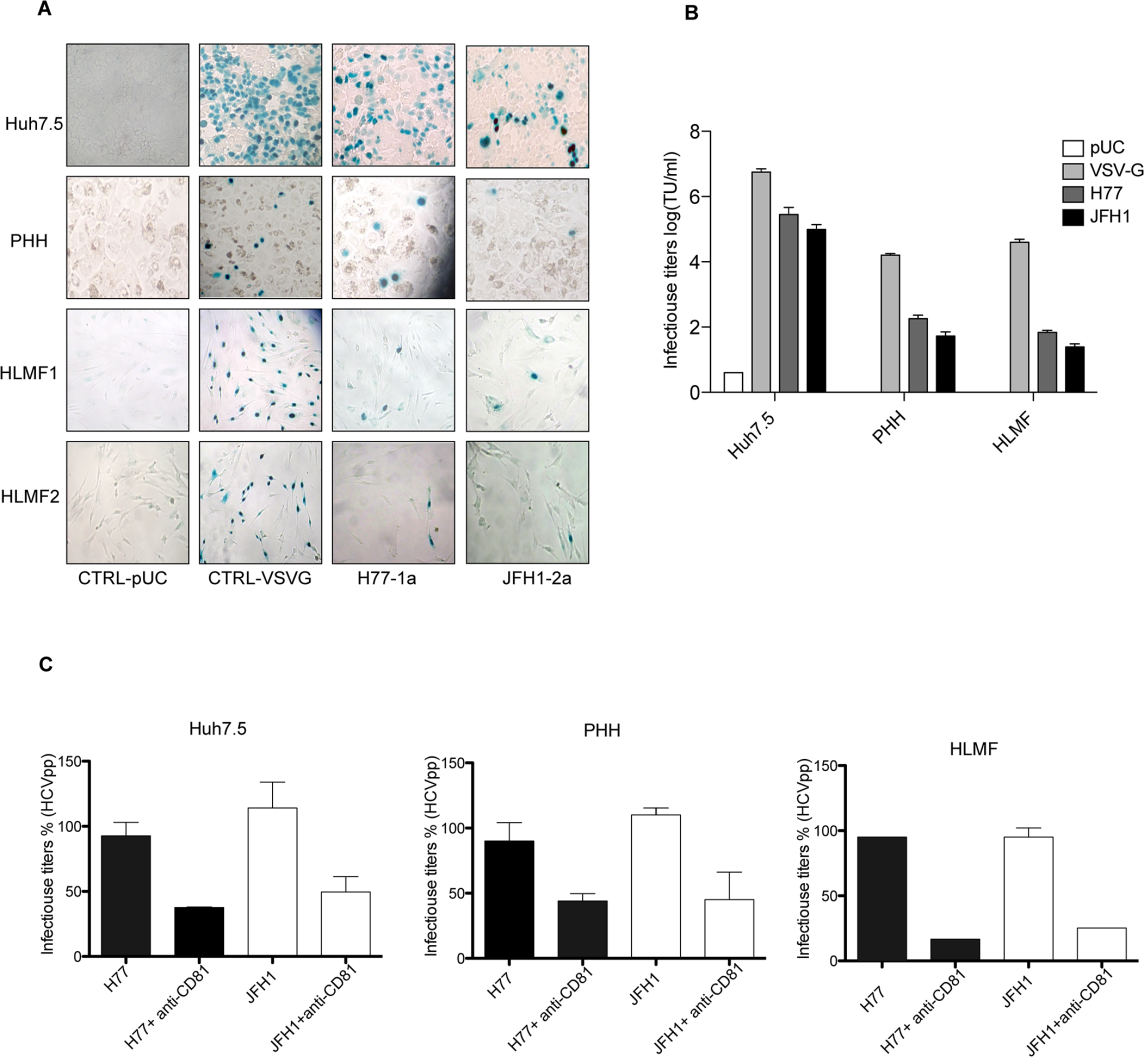
Permissiveness of HLMF to HCVpp. Huh7.5, PHH and HLMF were infected with HCVpp (JFH-1a or H77-a), VSVGpp or CTRL-PUC (pUC19 no envelops). **(A)** The Huh7.5, PHH and HLMF were infected with HCVpp of genotype (1a) or (2a) carrying a lacZ marker gene encoding a nuclear-targeted ß-galactosidase. The expression of LacZ in cultured cells was performed with X-Gal staining after 72h (the nuclei of positive cells are stained blue) and shown by optical microscopy (magnification x20). **(B)** Histograms represent the infectious titers of HCVpp per ml (TU/ml) (means ± SD, two cell preparations). **(C)** Effect of anti-CD81 on HCVpp entry. The different types of cells were preincubated with anti-CD81 or irrelevant isotype control for 1h at 37°C, followed with inoculation of HCVpp for 72h and the expression of LacZ was measured by X-Gal staining. Data represent a percent of neutralization of infectious titers HCVpp.

To examine HCVpp entry, we used pseudoparticles harboring the VSV-G protein as a positive control and no-envelope pseudoparticles, as a negative control. HCVpp of genotypes (2a and 1a) were used to infect HLMF, Huh7.5 and PHH. The cells infected with HCVpp were subjected to an X-gal staining procedure at 72h after infection. As shown in Fig 3A and 3B, HCVpp from genotypes 1a and 2a readily infected Huh7.5 cells. PHH and HLMF were permissive for HCVpp entry at a lower level compared to Huh7.5. The positive control VSV-G infected easily Huh7.5 and HLMF but lowly PHH (Fig 3A and 3B). The pseudoparticles lacking glycoproteins were used as a negative control and did not infect the different cells used in this study. These results demonstrated that HCVpp could infect HLMF.

### Neutralization of HCVpp entry by anti-CD81 antibody

To investigate the receptor dependency of cell infection, we assessed the ability of an antibody specific of CD81 to inhibit HCVpp-h77 or HCVpp-JFH1 infection (Fig 3C). Anti-CD81 mAb reduced HCVpp-H77 and HCVpp-JFH1 infection of Huh7.5, PHH and HLMF cells by more than 50%, demonstrating that infection was CD81-dependent. In contrast, the anti-CD81 had not effect on cells infected with VSV-G (Data no shown). The percent neutralization was calculated relative to the irrelevant IgG control.

### Infection of HLMF by HCVcc

**Fig 4:**
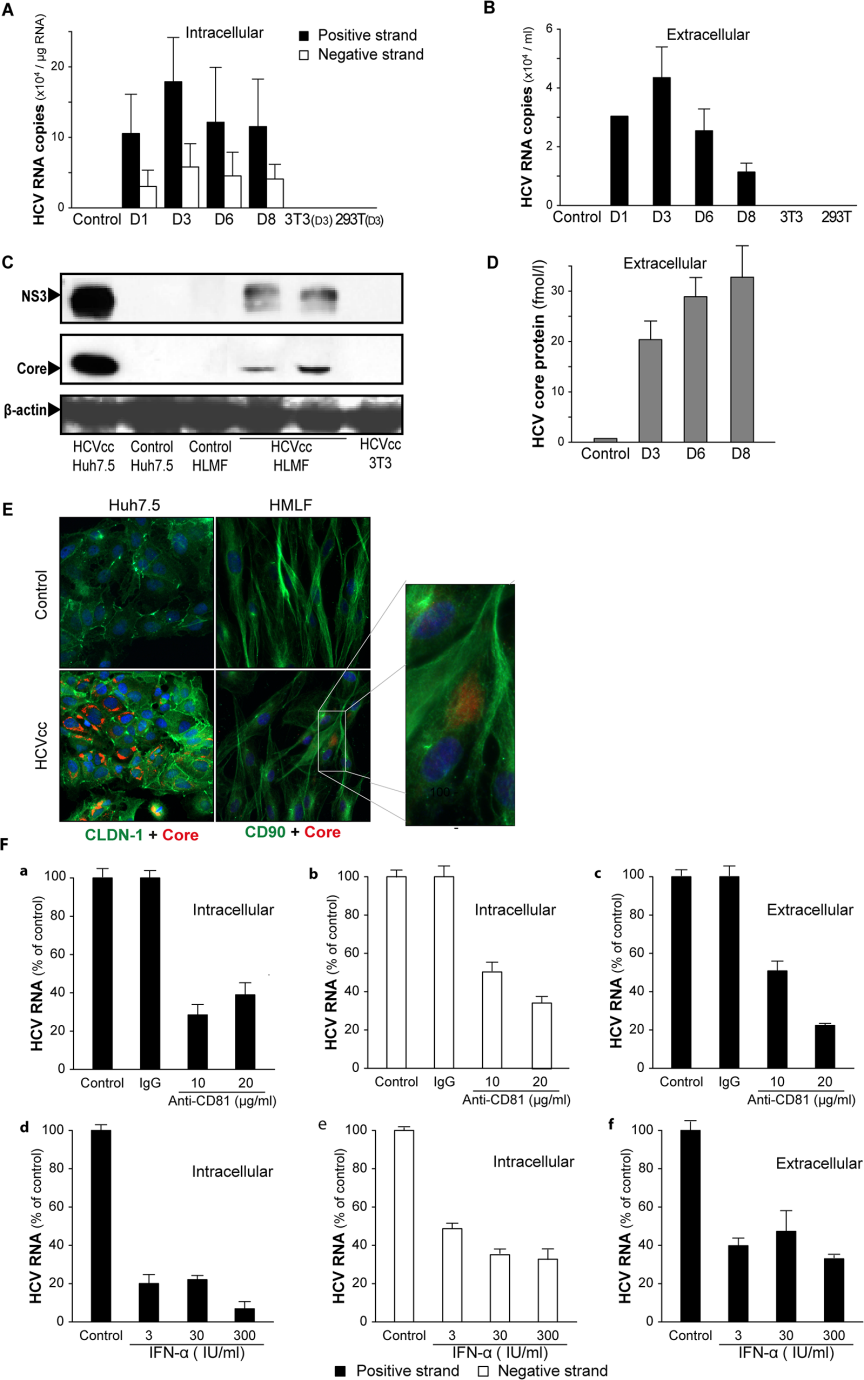
Infection status of HLMF inoculated with HCVcc. HLMF were inoculated with JFH1-HCVcc 24 hours after plating. Parameters of HCV infection were monitored at the indicated days after inoculation, and in non-infected HLMF or 3T3 and 293T cells inoculated with JFH1-HCVcc, as controls. Strand-specific HCV RNA was measured by RT-PCR: **(A)** In lysed cells, **(B)** In filtered culture supernatants. Histograms represent the copies of strand-specific HCV RNA per μg of total cellular RNA or per ml of supernatant (means ± SD, Ten cell preparations). **(C)** Intracellular levels of HCV NS3 and core proteins were analyzed by Western blot after 72h post infection in comparison with Huh7.5 or 3T3 cells inoculated with JFH1-HCVcc and with non-infected cells. **(D)** Levels of HCV core protein in filtered culture supernatants were measured by chemiluminescent microparticle immunoassay (means ± SD, two cell preparations). The blots and histograms are representative of seven HLMF preparations. **(E)** Immunofluorescence on HLMF with co-staining (CD90 and HCV core protein) and on Huh7.5 with co-staining (CLDN-1 and HCV core protein). **(F)** Inhibition of HCVcc infection by anti-CD81 antibody or IFN-lpha in HLMF. HLMF were incubated with (a-c) an anti-CD81 neutralizing monoclonal antibody or an isotype-matched control antibody, added 1 h before HCVcc inoculation, or with (d-f) IFN-lpha or a vehicle after HCVcc inoculation. The concentrations tested are indicated. HCV infection was evaluated three days after the inoculation by RT-PCR analysis of strand-specific HCV RNA (a, b, d, e) in lysed cells, and (c and f) in filtered culture supernatants. Histograms represent the copies of strand-specific HCV RNA per μg of total cellular RNA or per ml of supernatant, from triplicate independent experiments.

To determine whether HCV genome replication could occur in HLMF, we inoculated these cells with HCVcc. After the HCVcc challenge, positive-strand HCV RNA and negative-strand HCV RNA, a hallmark of HCV genome replication, were both detected in these cells, as shown using a strand-specific quantitative RT-PCR technique that was previously established [24]. The intracellular levels of negative- and positive-strand HCV RNA both increased until day 3 post-inoculation and then decreased (Fig 4A). In HLMF inoculated with UV-inactivated HCVcc, the levels of positive-strand HCV RNA were reduced by more than 95% on day 3 post-inoculation and were undetectable thereafter, whereas negative-strand HCV RNA was undetectable at all time points (data not shown). Neither strand was detectable in HCVcc-inoculated 3T3 or 293T cells (Fig 4A).

We then attempted to determine whether viral particles were produced by infected HLMF. While negative-strand HCV RNA was consistently absent from filtered culture supernatants, positive-strand HCV RNA was detected from day 1 to day 8 post-inoculation (Fig 4B). In addition to HCV replication, we were able to show that HCV core (structural) and NS3 (non structural) proteins were produced by infected HLMF (Fig 4C and 4D). Although less abundant than in Huh7.5 cells infected with HVCcc, the two proteins were readily detected by Western blot in infected HLMF (Fig 4C). Neither protein was detected in control non-infected cells or in 3T3 fibroblasts subjected to the same challenge with HCVcc. Furthermore, HCV core protein was detected in the culture supernatants of infected HLMF and its concentration increased over time in these supernatants (Fig 4D).

However, the supernatants were not infective for naïve Huh7.5 cells, as determined by in situ immunofluorescence [28] (data not shown). The absence of production of infective particles by HLMF could explain the decrease of RNA level over time (Fig 4B). In contrast, Huh7.5 and PHH were infected by HVCcc (S1 Text) and (S3 Fig).

In addition, HCV core was detected in HLMF co-stained for CD90 and HCV core protein (Fig 4E), demonstrating that HLMF support HCV infection and replication. As anticipated, most Huh7.5 were positive for HCV core.

### Inhibitory effects of anti-CD81 antibody and IFN-α on HLMF infection by HCVcc

Increasing evidence has indicated that CD81 is essential for the entry of HCV into cells. In HLMF pre-treated with anti-CD81 neutralizing antibodies, the intracellular accumulation of both positive and negative HCV RNA strands was markedly reduced (by 75% and 50%, respectively) (Fig 4F-a and 4F-b). Anti-CD81 pre-treatment also caused a marked reduction in extracellular levels of positive-strand HCV RNA (Fig 4F-c). These results indicate that HCVcc replication in HLMF required an interaction between HCV envelope glycoprotein(s) and CD81. The isotype-matched control antibody exerted no inhibitory effect on intracellular levels of positive or negative-strand HCV RNA or on extracellular levels of HCV RNA (Fig 4F, a-c).

In the present study, we found that treating HLMF with IFN-a led to a fall in the intracellular levels of both positive- and negative-strand HCV RNA (Fig 4F-d and-e) and in extracellular levels of positive-strand HCV RNA (Fig 4F-f). The inhibitory effects of anti-CD81 antibody and IFN-a were also found in parallel in Huh7.5 and PHH infection by HCVcc (S1 Text) and (S4 Fig).

### HCVcc-induced pro-fibrotic changes in HLMF

**Fig 5:**
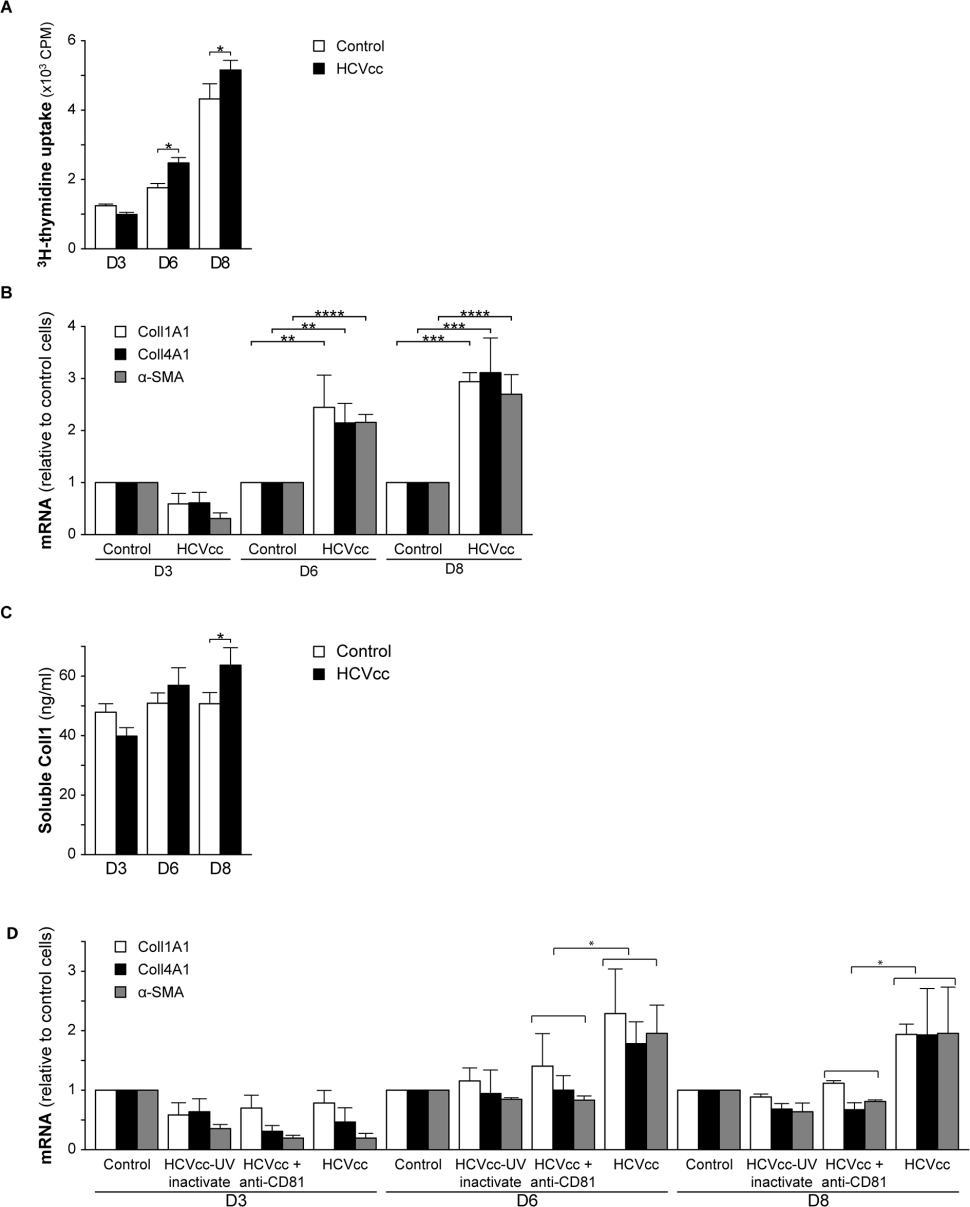
HCVcc-induced profibrotic changes in HLMF. HLMF were inoculated with JFH1-HCVcc 24 hours after plating, and analyzed at the indicated days after inoculation, together with non-infected cells: **(A)** Cell proliferation was measured by [^3^H]-thymidine incorporation. **(B)** The expression of a-SMA, collagen 1 and collagen 4 was estimated by RT-PCR, and expressed as mRNA levels relative to those in non-infected cells. **(C)** Secretion of the C-terminal propeptide of collagen I was measured by enzyme immunoassay in cell supernatants. The results are means ± SD of at least seven cell preparations, * p≤0.04, ** p = 0.01, *** p = 0.001 and **** p<0.0001 *vs*. control. **(D)** Role of HCVcc infection and replication on HLMF activation and collagen production. Non-infected HLMF, and HLMF inoculated with JFH1-HCVcc, together with UV-inactivated HCVcc or HCVcc treated with anti-CD81 for 24 hours after plating, were cultured and analyzed at the indicated days after inoculation. The relative expression of a-SMA, collagen 1 and collagen 4 was estimated by RT-PCR, and expressed as mRNA levels relative to those in non-infected cells. The results are means ± SD of three cell preparations, * p≤0.05, *vs*. control.

Cell proliferation tended to decrease in HLMF three days after the HCVcc challenge, but thereafter became significantly higher than in control non-infected cells (Fig 5A). The transcript levels of a-SMA and collagens I and IV did not differ significantly between infected and non-infected cells on day 3, and thereafter were significantly higher in infected cells than in non-infected cells, by more than 2-fold on day 6, and by 2.5- to 3-fold on day 8 (Fig 5B). Increased collagen I production also resulted in higher levels of its C-terminal propeptide in the culture supernatants of HCVcc-infected cells (56.36±5.87 ng/ml and 63.70±5.83 ng/ml *versus* 50.84±3.4 ng/ml and 50.72±3.78 ng/ml in non-infected cells, after 6 and 8 days, respectively) (Fig 5C). HCV infection had no effect on cell viability (S2 Fig). We conclude from these findings that the HCVcc infection of HLMF increased cell proliferation, myofibroblastic differentiation and extracellular matrix production.

To better establish the link between the infection of HLMF by HCV and profibrotic properties, we compared the relative expression of a-SMA, and collagens I and IV mRNAs in cells infected with either HCVcc or UV-inactivated HCVcc, and in HCVcc-infected cells treated with anti-CD81. The relative expressions of a-SMA and collagens I and IV did not differ in the different treatment groups on day 3. After approximately one week (on days 6 and 8), the levels of a SMA and collagens I and IV increased in HCVcc-infected cells by approximately 2-fold when compared with UV-inactivated HCVcc-infected cells, and this increase was limited by anti-CD81 treatment (Fig 5D). These results show that the extracellular exposure of HLMF to inactivated HCVcc or HCV receptor blocking antibodies impairs myofibroblast activation and collagen production. The infection of HLMF by HCVcc is thus mandatory for this direct pro-fibrotic effect of the virus to occur.

### Sample variability

HLMF were isolated from 15 patients with normal liver, and 12 of these 15 preparations (80%) could be infected by HCVcc, as demonstrated by the presence of positive and negative-strand HCV RNA. All of infected HLMF were activated and produced collagen, although in one case the level of production was very low.

### Infection status of HLMF from HCV-infected patients

**Fig 6:**
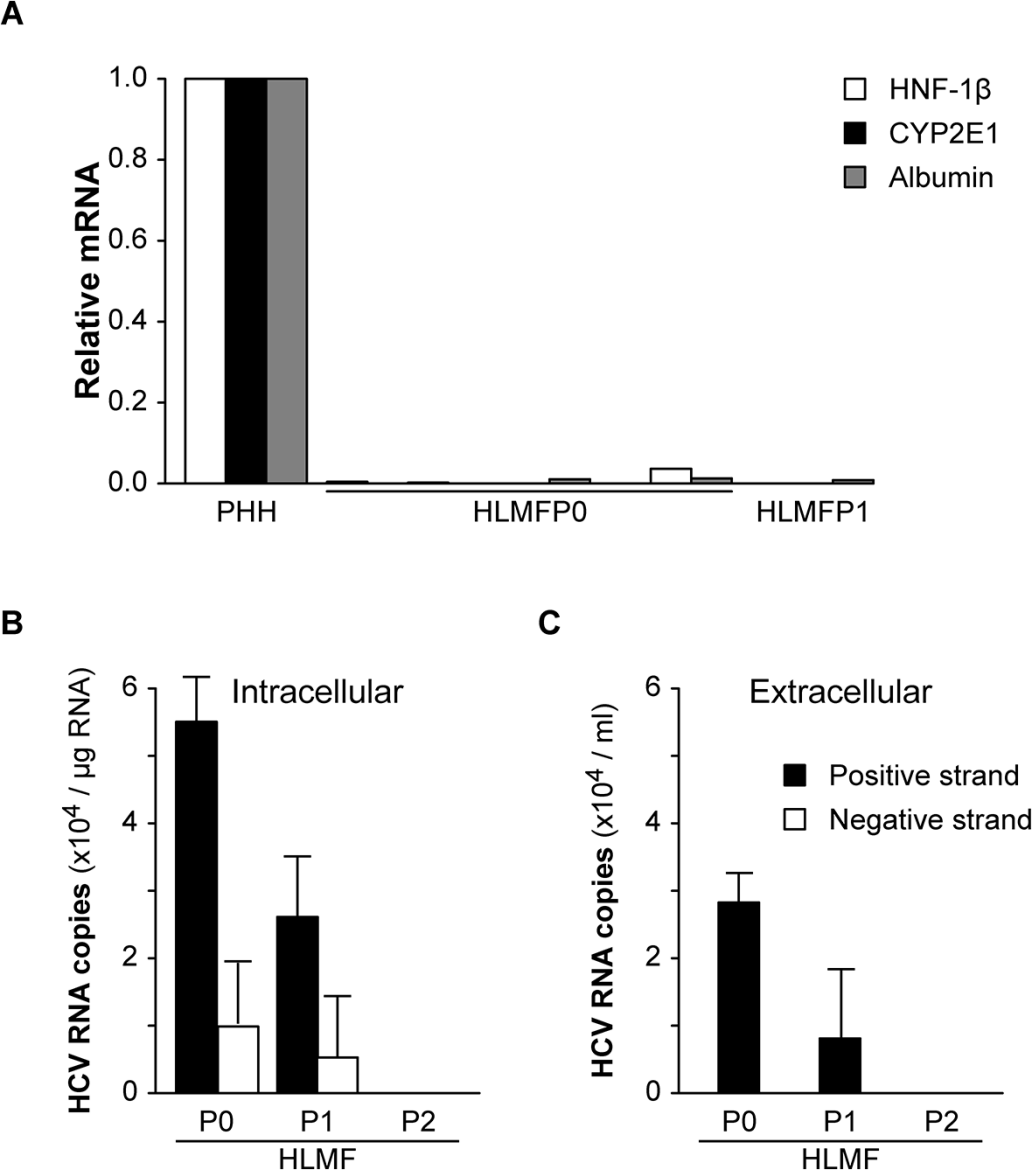
Infection status of HLMF from HCV-infected patients. HLMF were prepared from HCV-infected subjects and monitored after 7 days of primary culture (P0) and after 1 or 2 passages (P1, P2): **(A)** to ascertain the absence of hepatocyte contamination using RT-PCR analysis of HNF-1ß, CYP2E1 and albumin. The results are expressed as mRNA levels in individual HLMF preparations relative to those in primary cultures of human hepatocytes (PHH); to detect HCV infection using RT-PCR analysis of strand-specific HCV RNA **(B)** in lysed cells and **(C)** in filtered culture supernatants. Histograms represent the copies of strand-specific HCV RNA perμg of total cellular RNA or per ml of supernatant, from two cell preparations.

HLMF were prepared from HCV-infected subjects in order to examine whether these cells were infected *in vivo*. Cells from HCV-infected patients have been used only in this experimentation. In primary culture and up to at least two passages, these cell preparations expressed the markers of mesenchymal cells (vimentin), fibroblasts (CD90) and myofibroblasts (a-SMA) (data not shown). Hepatocyte contamination was excluded using RT-PCR to detect albumin, CY2E1 and HNF-1ß mRNAs, both well-established hepatocyte markers [12], which were undetectable in all the preparations tested (Fig 6A). Furthermore, during cell characterization by flow cytometry, anti-hepatocyte specific markers were consistently negative (data not shown).

These cell preparations contained positive-strand and negative-strand HCV RNA at the times of primary culture (Passage 0, P0) and after one passage (P1) (Fig 6B). At subsequent passages, both strands became undetectable (Fig 6B). Positive-strand HCV RNA was also detected in the culture supernatants of HLMF from HCV-infected patients at the times of primary culture (P0) and at P1 (Fig 6C).

## Discussion

The mechanisms of hepatic fibrogenesis caused by HCV infection are not fully understood. HCV-replicating hepatocytes synthesize increased amounts of TGF-ß, which induces a fibrogenic response in liver myofibroblasts [6]. Bataller *et al*. reported that HCV core protein, and the nonstructural proteins NS3 and NS5, which were transduced into hepatic stellate cells by adenoviral vectors, exerted direct profibrogenic effects [17]. It has also been reported that a direct interaction occurs between the HCV envelope glycoprotein E2 and CD81 in human hepatic stellate cells and induces an up-regulation of MMP-2 expression [29].

JFH1-HCVcc has already been shown to replicate in cells other than hepatocytes. In particular, based on the detection of HCV proteins and of positive and negative strands of HCV RNA, it has been shown that the JFH1-HCV subgenomic replicon was able to replicate in mouse embryonic fibroblasts [30] and in a human hepatic stellate cell line [31]. Furthermore, the JFH1-HCV subgenomic replicon can establish and maintain replication in embryonic fibroblasts, with a stable expression of viral proteins and viral RNA [32].

In a recent study, Florimond *et al* suggested that HSC were not permissive for HCV entry [33]. However, they mostly used the immortalized cell line LX-2, and three preparations of myofibroblast obtained by outgrowth from liver explants in their experiments. These latter cells might represent a particular subpopulation of HLMF that lack molecules of the HCV “receptor complex Moreover”, the myofibroblasts in their work were used after 3 and up to 7 passages, culture stages at which we found the expression of several receptor molecules, such as OCLN or LDLR, was decreased or even lost. In the present study, by contrast, we used a culture model of isolated cells, representative of the mixed population of myofibroblasts including HSC-derived myofibroblasts that arise in the human fibrotic liver. Using cell preparations at earlier stage (*i*.*e*. passage 2), we could demonstrate that HLMF expressed all factors of the HCV entry complex and could be infected with HCVpp and JFH1-HCVcc. We also demonstrated that HCVcc stimulated these cells to produce ECM.

Our data indicate that HLMF support HCVpp entry, and that this infection can be inhibited by a specific antibody of CD81 receptor. The detection of positive-strand HCV RNA by itself does not prove HCV replication, because viral RNA may be bound or taken up without undergoing a complete infectious cycle [34]. It is therefore noteworthy that we were also able to detect negative-strand HCV RNA in infected HLMF, thus confirming viral replication. This detection was made possible by the sensitive method used in this work, and previously developed in our group [24]. The high level of viral load observed in Huh7.5 cell line compared with HLMF at 3 days post-infection could be explained by the higher expression of the receptor molecules in this cell line. Further evidence for autonomous HCV replication in HLMF was provided by the detection of intracellular NS3 and core protein, and by the increasing levels of core protein in the culture supernatant. In this work, we were not able to show that infected HLMF produced infectious viral particles, unlike infected human hepatocytes [12], suggesting that replication occurs at lower levels in these cells than in hepatocytes, the major reservoir of the virus in the organism determined so far, or that infection might be defective in HLMF, without any production of infective particles.

CD81 plays a critical role in an early step of normal primary human hepatocyte infection by HCV. Molina et al. reported that the infection of primary hepatocytes with serum from HCV-infected patients could be inhibited by anti-CD81 neutralizing antibodies [35]. Here, we found that both anti-CD81 neutralizing antibodies and IFN-alpha efficiently reduced the levels of positive- and negative-strand HCV RNA in HCVcc-infected HLMF.

HCVcc infection also induced pro-fibrotic phenotypic changes in HLMF, as shown by increased cell proliferation, alpha-SMA expression and collagen production. Tan et al. showed that recombinant HCV proteins did not induce any direct pro-fibrogenic effect on HLMF [36]. In contrast, infection by HCV induced HLMF activation and collagen production. Indeed, we also observed that extracellular exposure to HCV was not sufficient to induce HLMF activation and collagen production, providing evidence that HCV infection is necessary to induce a fibrogenic effect. Importantly, the clinical relevance of these mechanisms was supported by the demonstration that HLMF derived from patients with chronic hepatitis C were also infected by HCV.

The current findings highlight an alternative pathway of liver fibrosis in chronic HCV infection, which may be important to consider, especially when liver inflammation is limited or absent. These data provide a new physiopathological aspect of HCV-driven fibrogenesis, in which myofibroblasts are the center of the process. These findings also provide a model to test new drugs that could be further developed in patients with a rapidly evolving fibrogenesis process.

## Supporting Information

S1 Fig Expression of HCV receptors in HLMF at different passages. Quantitative RT–PCR. Histograms represent mRNA levels of CD81, LDLR and OCLN expression from 5 HLMF preparations with at least 4 different passages (means ± SD).
(TIF)

S2 Fig Effect of HCVcc infection on cell viability in HLMFs. HLMFs were inoculated with JFH1-HCVcc 24 hours after plating. Cell death was monitored by flow cytometry at the indicated days after inoculation, and in HLMFs that were not challenged or treated with C2-ceramide, as a positive control for apoptosis. The percentage of A) apoptotic cells, defined as annexin V-positive, propidium iodide-negative cells; B) necrotic cells, defined as propidium iodide-positive cells, were determined in duplicate experiments.
(TIF)

S3 Fig Infection status of Huh7.5 and PHH inoculated with HCVcc. (A): Huh7.5 (a, b, c) and (B): PHH (d, e, f) were inoculated with JFH1-HCVcc 24 hours after plating. The parameters of HCV infection were monitored at the indicated days after inoculation, and as in non-infected cells, strand-specific HCV RNA was measured by RT-PCR: (A-a, A-b, B-a and B-b) in lysed cells, (A-c and B-c) in filtered culture supernatants. Histograms represent the copies of strand-specific HCV RNA perμg of total cellular RNA or per ml of supernatant. (C): Cells were lysed at 72h post infection and the expression of core protein and NS3 were analyzed using Western blot analysis.
(TIF)

S4 Fig Inhibition of HCVcc infection by anti-CD81 antibody or IFN- in Huh7.5 and PHH. Huh7.5 (A) and PHH (B) were incubated with (a, b, c) an anti-CD81 neutralizing monoclonal antibody or an isotype-matched control antibody, added 1 h before HCVcc inoculation, or with (d, e, f) IFN-lpha or a vehicle after HCVcc inoculation. The concentrations tested are indicated. HCV infection was evaluated three days after inoculation by RT-PCR analysis of strand-specific HCV RNA (a, b, d, e) in lysed cells, (c, f) in filtered culture supernatants. Histograms represent the copies of strand-specific HCV RNA per μg of total cellular RNA or per ml of supernatant, from duplicate experiments.
(TIF)

S1 Text Supplementary results and supplementary methods. (DOC)
