# Supplementary figures and images for: Infection of Human Liver Myofibroblasts by Hepatitis C Virus: A Direct Mechanism of Liver Fibrosis in Hepatitis C
Source: PLoS One. 2015 Jul 27;10(7):e0134141. doi: 10.1371/journal.pone.0134141 (PMC4516308; doi:10.1371/journal.pone.0134141)

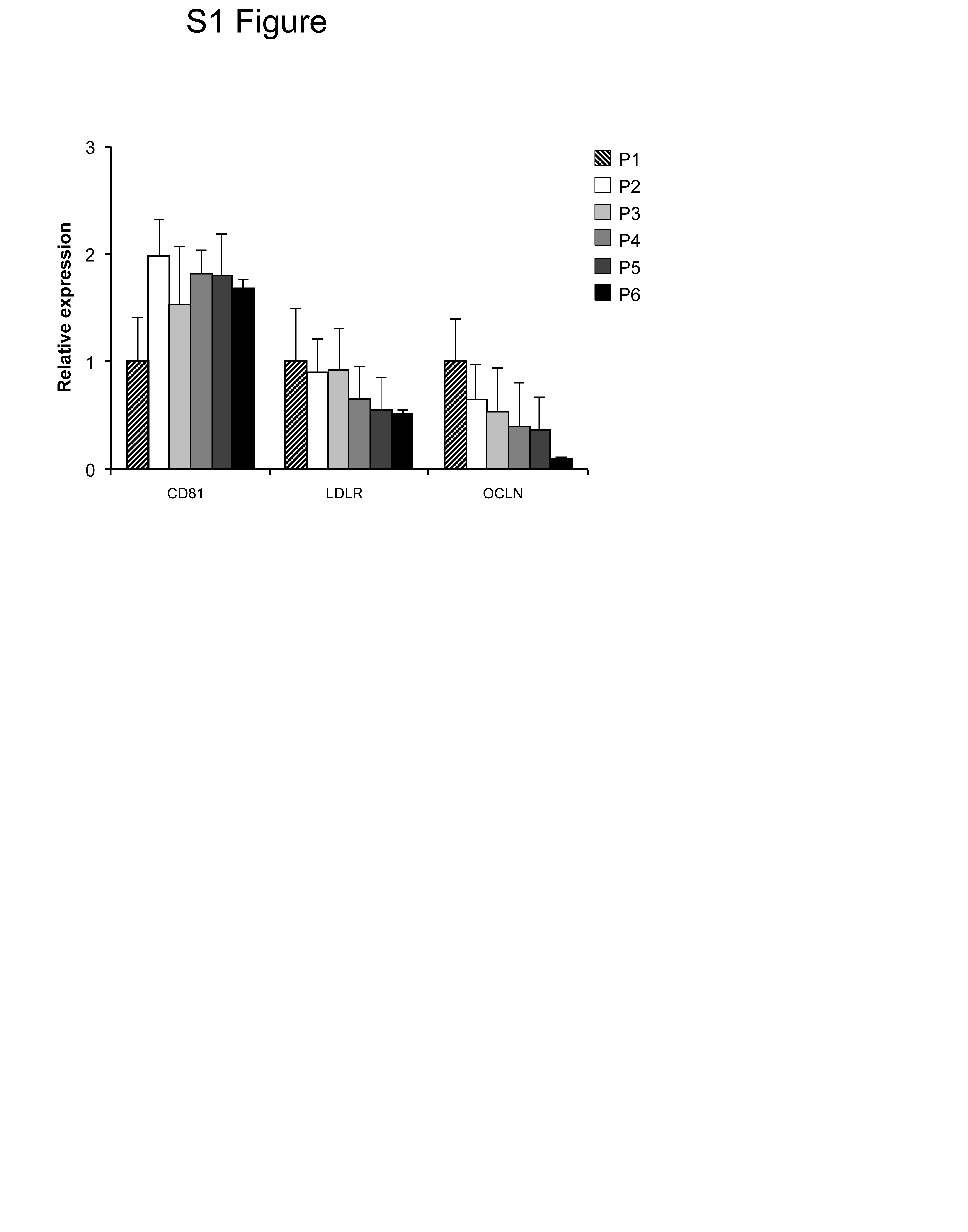

Supplement: S1 Fig — Quantitative RT–PCR. Histograms represent mRNA levels of CD81, LDLR and OCLN expression from 5 HLMF preparations with at least 4 different passages (means ± SD). (TIF) [file pone.0134141.s001.tif]

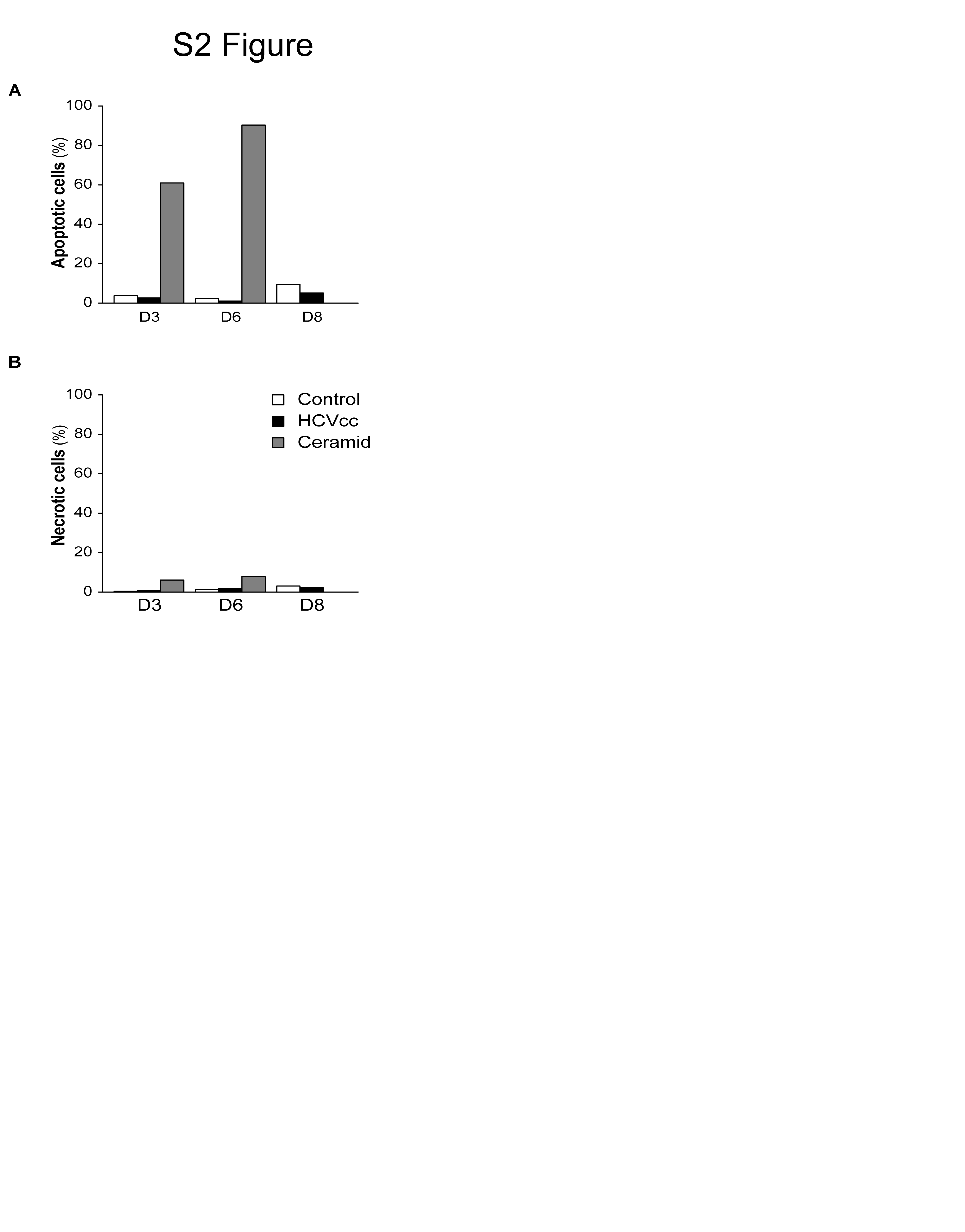

Supplement: S2 Fig — HLMFs were inoculated with JFH1-HCVcc 24 hours after plating. Cell death was monitored by flow cytometry at the indicated days after inoculation, and in HLMFs that were not challenged or treated with C2-ceramide, as a positive control for apoptosis. The percentage of A) apoptotic cells, defined as annexin V-positive, propidium iodide-negative cells; B) necrotic cells, defined as propidium iodide-positive cells, were determined in duplicate experiments. (TIF) [file pone.0134141.s002.tif]

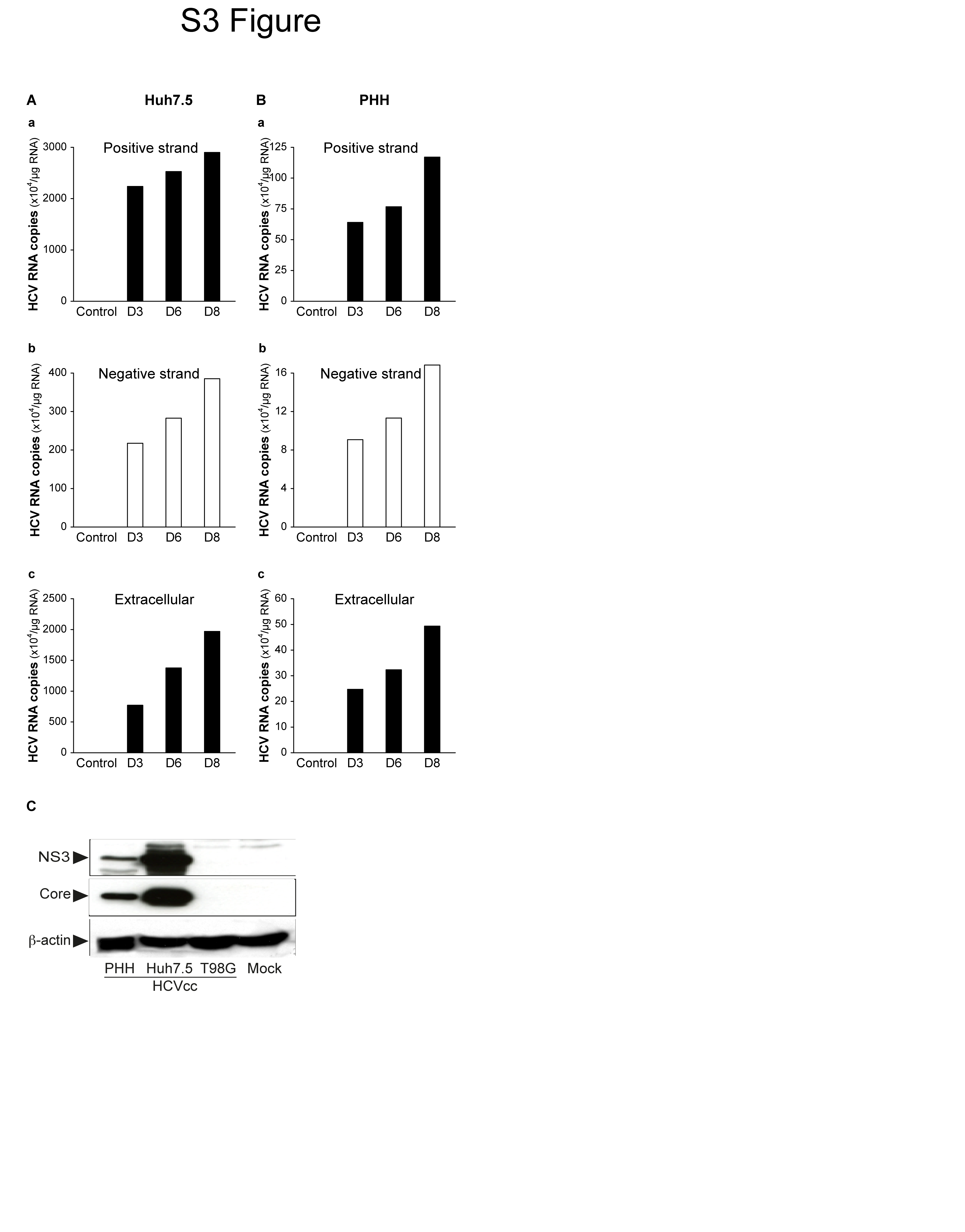

Supplement: S3 Fig — (A): Huh7.5 (a, b, c) and (B): PHH (d, e, f) were inoculated with JFH1-HCVcc 24 hours after plating. The parameters of HCV infection were monitored at the indicated days after inoculation, and as in non-infected cells, strand-specific HCV RNA was measured by RT-PCR: (A-a, A-b, B-a and B-b) in lysed cells, (A-c and B-c) in filtered culture supernatants. Histograms represent the copies of strand-specific HCV RNA perμg of total cellular RNA or per ml of supernatant. (C): Cells were lysed at 72h post infection and the expression of core protein and NS3 were analyzed using Western blot analysis. (TIF) [file pone.0134141.s003.tif]

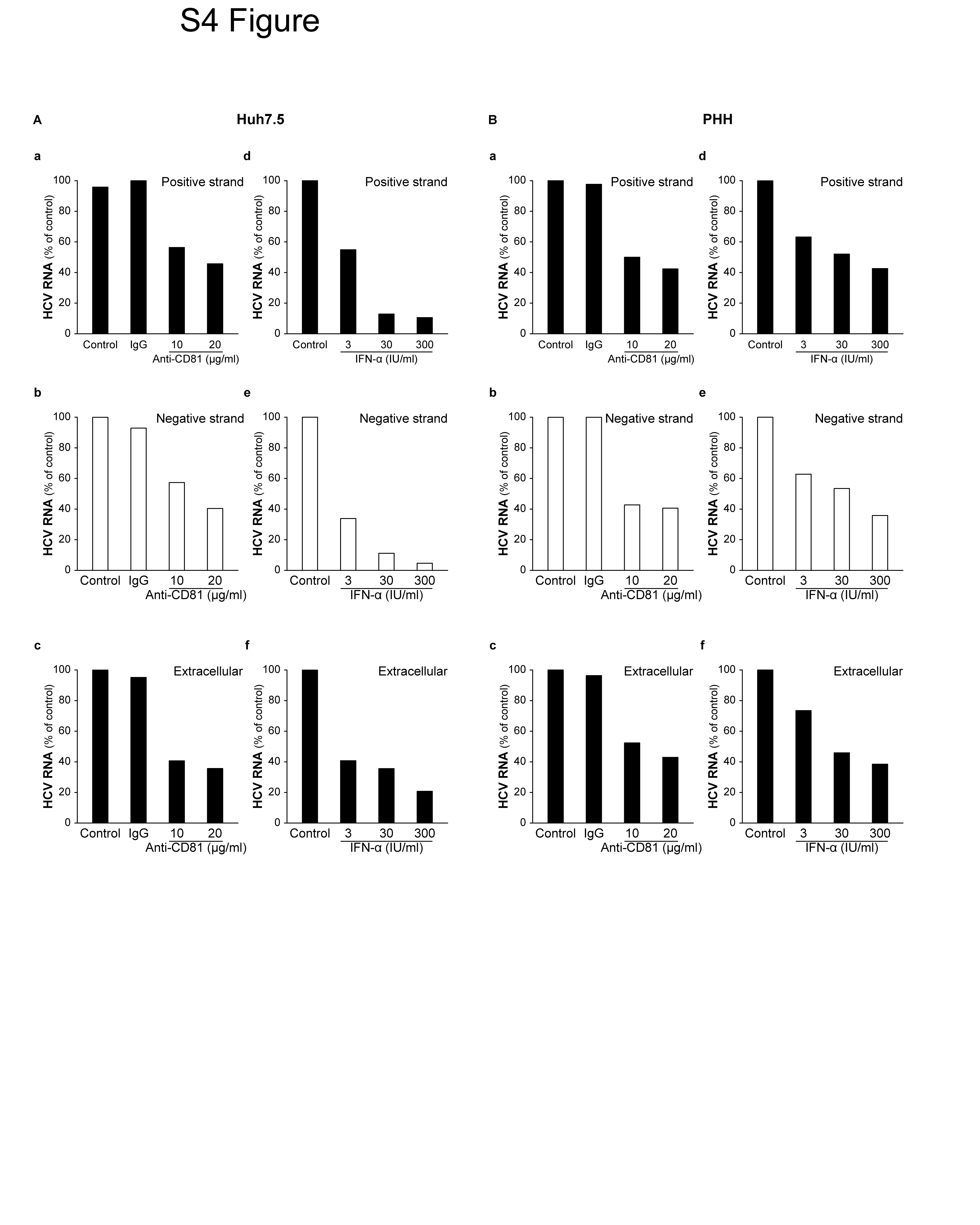

Supplement: S4 Fig — Huh7.5 (A) and PHH (B) were incubated with (a, b, c) an anti-CD81 neutralizing monoclonal antibody or an isotype-matched control antibody, added 1 h before HCVcc inoculation, or with (d, e, f) IFN-lpha or a vehicle after HCVcc inoculation. The concentrations tested are indicated. HCV infection was evaluated three days after inoculation by RT-PCR analysis of strand-specific HCV RNA (a, b, d, e) in lysed cells, (c, f) in filtered culture supernatants. Histograms represent the copies of strand-specific HCV RNA per μg of total cellular RNA or per ml of supernatant, from duplicate experiments. (TIF) [file pone.0134141.s004.tif]
