# Supplementary material for: Infection of Human Liver Myofibroblasts by Hepatitis C Virus: A Direct Mechanism of Liver Fibrosis in Hepatitis C
Source: PLoS One. 2015 Jul 27;10(7):e0134141. doi: 10.1371/journal.pone.0134141 (PMC4516308; doi:10.1371/journal.pone.0134141)
Supplement: S1 Text — (DOC) [file pone.0134141.s005.doc]

**Supplementary results**

**Evaluation of the effect of HCV on the apoptosis and necrosis of HLMFs**

We first determined the potential effects of HCV infection on cell viability. After HCVcc infection, HLMFs displayed minimal apoptosis when compared with that induced by C2-ceramide treatment (S2-A Fig), and minimal necrosis (S2-B Fig). Thus, no significant toxicity resulted from HCVcc infection in HLMFs.

# Infection of Huh7.5 and PHH by HCVcc

In parallel with determining HCV genome replication in HLMFs, and as a positive control for HLMFs infection, Huh-7.5 (S3-A Fig) and PHH (S3-B Fig) were also inoculated with HCVcc, intracellular positive and negative-strand HCV RNA, and extracellular positive-strand HCV RNA was evaluated using quantitative RT-PCR. As anticipated, all HCV RNAs were detected at high levels, which also rose 8 days after inoculation in both cell types. Using Western blot analysis, the expression of core protein could also be detected in Huh7.5 and PHH infected with HCVcc, but could not be detected in T98G cell line (S3-C Fig).

**Inhibitory effects of anti-CD81 antibody and IFNα on Huh7.5 and PHH infection by HCVcc**

The inhibitory effects of anti-CD81 antibody and IFNα on the infection of Huh7.5 (S4-A Fig) and on PHH (S4-B Fig) with HCVcc were determined as a positive control. As anticipated, and as with infected HLMFs, in both cell types, anti-CD81 and IFNα induced a marked reduction of intracellular positive and negative HCV RNA strands and of extracellular levels of positive-strand HCV RNA. The isotype-matched control antibody had no inhibitory effect on intracellular levels of positive and negative-strand HCV RNA or on extracellular levels of HCV RNA.

**Supplementary Methods**

**Cell death assay**

Cell death was analyzed by flow cytometry using the annexin V-FITC kit (Immunotech, Marseille, France) and propidium iodide (PI) staining to detect apoptosis and necrosis, respectively. Cell treatment with 20 μM of C2-ceramide (Sigma, Fallavier, France) was used to induce apoptosis, as a positive control. Five thousand cells were analyzed in each sample. Annexin V-positive, PI-negative cells were considered as apoptotic, while PI-positive cells were deemed to be necrotic and unstained cells viable**.**

**HCV inoculation and inhibition in Huh7.5 and PHH**

JFH1-HCVcc stocks were used to inoculate Huh7.5 for 24 hours and PHH for 72 hours after plating, at a MOI of 0.1 ffu per cell. After a 12-hour incubation at 37°C, the inoculum was removed and the cells were washed three times with phosphate-buffered saline. The cultures were then continued in complete medium for a maximum of 8 days.

For inhibition studies, Huh7.5 and PHH were pre-incubated with the same concentrations of an anti-CD81 neutralizing monoclonal antibody used in HLMFs or an isotype mouse IgG1, k (Both from BD Farmagen,) for 1 h before HCVcc inoculation, or interferon-alpha (IFN-α) after HCVcc inoculation. HCV infection was evaluated three or eight days after the challenge.
